# Pre-treatment patient selection for nivolumab benefit based on serum mass spectra

**DOI:** 10.1186/2051-1426-3-S2-P103

**Published:** 2015-11-04

**Authors:** Jeffrey Weber, Alberto J Martinez, Heinrich Roder, Joanna Roder, Krista Meyer, Senait Asmellash, Julia Grigorieva, Maxim Tsypin, Carlos Oliveira, Arni Steingrimsson, Kevin Sayers, Antonella Bacchiocchi, Mario Sznol, Ruth Halaban, Harriet Kluger

**Affiliations:** 1 H. Lee Moffitt Cancer Center, Tampa, FL, USA; 2 Biodesix Inc., Boulder, CO, USA; 3 Yale University School of Medicine, New Haven, CT, USA; 4 Department of Medical Oncology, Yale Cancer Center, New Haven, CT, USA

## Introduction

The durability of anti-tumor responses observed in patients treated with antibodies blocking PD-1 has provided a central role for these drugs in melanoma therapeutics. Identifying predictive biomarkers to aid therapeutic decision making is critical for realizing the full potential of these immunotherapies. We report on the development of a pre-treatment serum test to separate melanoma patients into two groups with significantly different outcomes following nivolumab therapy.

## Methods

Pre-treatment serum samples were available from 119 patients in the NCT01176461 study (“SET1”) and 30 patients from an observational study (“SET2”) at two institutions. All patients had advanced un-resectable melanoma and received nivolumab.

Mass spectra were collected from all samples using the “deep MALDI” approach [1]. We identified 351 mass spectral peaks for use in classifier construction. SET1 was split into a development (DEV) (N=60) and an internal validation (VAL) (N=59) set. Deep learning methods were used to construct a classifier correlating with time-to-event data in a fashion similar to Roder et al [2] using only the DEV set. This classifier was validated on the VAL set and a test was constructed using the same procedure with the whole SET1 and performance evaluated on the independent SET2.

## Results

The test separated the populations into two groups, “Early”/”Late”, with worse/better outcome on nivolumab treatment. The hazard ratios (HRs) between Early and Late groups are presented in Table 1. Test classification groups did not show any association with available PD-L1 expression data and remained significant in multivariate analysis.

**Table 1:** 

Set	HR(TTP)	Log-rank p-value	HR(OS)	Log-rank p-value
DEV	0.48	0.020	0.026	0.005
VAL	0.43	0.013	0.48	0.012
Full SET1	0.50	0.001	0.38	<0.001
SET2	n/a	n/a	0.26	0.002

## Conclusions

We have constructed a test to identify melanoma patients most likely to have improved survival on nivolumab therapy. The test validated in an independent sample set with HR~0.3 and appears to be independent of PD-L1 expression. Some proteins used in the test are related to acute Phase reactions and the complement system. While further validation and protein identification studies are needed, this test may become a clinically useful predictive biomarker for nivolumab therapy.

This research was supported in part by the Yale SPORE in Skin Cancer, funded by the NCI, NIH, under award number 1 P50 CA121974 (R.H.)

## References

[B1] Duncan M Proceedings of the 61st ASMS Conference on Mass Spectrometry and Allied Topics; June 8-9 2013 Minneapolis (MN) MP 181

[B2] Roder J Proceedings of the Annual Meeting of the AACR; Apr 1-5 2015 Philadelphia (PA) 5304

